# New Polymer–Carbon Lustrous Carbon Precursor in Synthetic Molding Sands—Part I: Studies on the Properties of Sands

**DOI:** 10.3390/ma17246054

**Published:** 2024-12-11

**Authors:** Beata Grabowska, Artur Bobrowski, Dariusz Drożyński, Dominika Kwaśniewska-Królikowska, Barbara Pilch-Pitera, Katarzyna Pojnar, Daniel Nowak

**Affiliations:** 1 Faculty of Foundry Engineering, AGH University of Krakow (AGH University), Reymonta 23, 30-059 Krakow, Poland; arturb@agh.edu.pl (A.B.); dd@agh.edu.pl (D.D.); 2 ZGM Zębiec S.A., 27-200 Starachowice, Poland; dominika.kwasniewska@zebiec.pl; 3 Department of Polymers and Biopolymers, Faculty of Chemistry, Rzeszow University of Technology, Powstańców Warszawy 6, 35-959 Rzeszów, Poland; barbpi@prz.edu.pl; 4 Doctoral School of Engineering and Technical Sciences, Rzeszow University of Technology, Powstańców Warszawy 12, 35-959 Rzeszów, Poland; d521@stud.prz.edu.pl; 5 Faculty of Mechanical Engineering, Wroclaw University of Science and Technology, Wybrzeże Wyspiańskiego 27, 50-370 Wroclaw, Poland; daniel.nowak@pwr.edu.pl

**Keywords:** foundry, cast, bentonite, montmorillonite, lustrous carbon, shungite, polyethylene

## Abstract

In the first part of this publication, selected technological and strength properties of synthetic molding sand bound with sodium bentonite with the addition of a new lustrous carbon carrier (R_c_^w^, R_m_^w^, R_k_, W_f_, P^w^, Z, P_D_, P_S_, S_LS_, ρ_0_) were determined. The introduction of polyethylene as a substitute for hydrocarbon resin, and shungite as a replacement for coal dust, demonstrated the achievement of an optimal molding sand composition for practical use in casting technology. The sand containing a new lustrous carbon carrier (SH/PE) demonstrates the highest permeability and flowability. Based on the analysis of the obtained results, it should be concluded that to achieve the desired level of the measured properties in the sand with the mixture of precursors SH/PE, the moisture content should be in the range of 1.5% to 1.7%.

## 1. Introduction

Synthetic molding sands (green sands) are crucial in casting processes, especially for cast iron production, ensuring high-quality castings with precise shapes and dimensions. These sands consist primarily of a mineral matrix (85–95% by weight, typically quartz sand), a binder (4–10%), and water (2–5%) [1]. The most commonly used binder, bentonite, is an aluminosilicate with a layered structure that adsorbs and absorbs water, enabling it to swell. Sodium activation enhances its sorption capacity, making sodium bentonite highly effective for improving mold quality [1,2,3,4,5,6,7,8,9].

**Figure 1:**
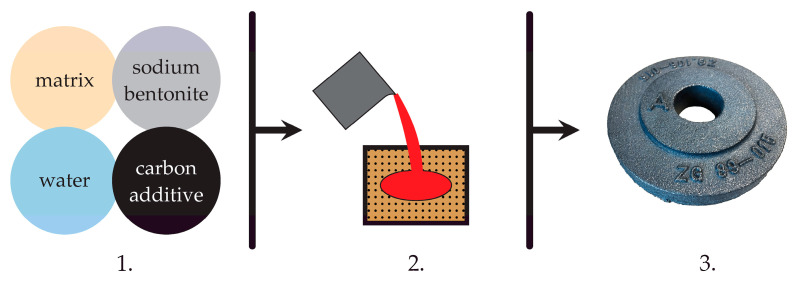
Diagram of the casting process: (**1.**) components of the molding sand constituting the building material of the mold, (**2.**) pouring the mold with liquid metal, (**3.**) casting.

To ensure a high-quality surface finish on cast iron castings, a precursor of lustrous carbon (lustrous carbon carrier), most commonly consisting of a mixture of hydrocarbon resin and carbonaceous materials in the form of carbon dust or graphite, is added to traditional bentonite-based molding sand (Figure 1) [1,10,11,12,13].

During thermal decomposition, this precursor produces a reducing gas phase, resulting in the formation of a pyrolytic carbon layer—a mixture of amorphous and lustrous carbon [10]. Additionally, the carbon additive enhances the technological properties of the molding sand, such as increasing the binding strength between matrix grains. The resulting lustrous carbon (LC) layer, approximately 1–10 μm thick, adsorbs onto the surface of the quartz matrix grains, forming a non-wettable barrier that prevents components from the liquid metal alloy from penetrating between the grains. This barrier inhibits high-temperature chemical reactions between the metal, non-metal oxides in the alloy, and the molding sand components. Consequently, the casting surface remains free from defects and other imperfections [14,15]. The amount of carbon additive used in the molding sand composition depends primarily on the carbon content and its capacity to form LC, typically ranging from 3% to 8%. Carbon additives have a theoretical capacity to form LC in a fairly wide range of 7–60%, but in practice the optimal level is generally in the range of 15–20% [16,17,18,19,20,21,22].

The thermal decomposition of carbon additives during the pouring of liquid metal into a wet mold releases a mixture of hydrocarbons, including harmful aromatic compounds and polycyclic aromatic hydrocarbons (PAHs). Commonly used carbon additives, particularly hydrocarbon resins, are associated with relatively high levels of atmospheric emissions of hazardous substances, such as aromatic hydrocarbons from the BTEX group (benzene, toluene, ethylbenzene, and xylenes) [18,23,24,25,26,27,28,29]. Additionally, excessive LC in the molding sand can lead to casting defects, including “metal discontinuities” and gaseous issues like “bubbles” and “pinholes”. Therefore, it is essential to carefully control both the quantity and quality of LC in the molding sand.

Proposed by the authors, the new lustrous carbon precursor consists of a two-component mixture of shungite (SH) and polyethylene (PE). Shungite is intended to replace the less environmentally friendly carbon dust (CD) commonly used in foundry applications. SH is a carbonaceous material found in rock deposits, characterized by a high carbon and silicon content and low emissions of harmful organic compounds during casting processes, especially compared with widely used carbonaceous additives like carbon dust [29,30,31,32,33,34,35]. The second component proposed by the authors for the new carbon precursor is polyethylene (PE), intended as a substitute for the hydrocarbon resins present in currently used carriers (e.g., HCR resin). PE is a linear polymer and, unlike resins, does not contain aromatic rings in its structure, making it inherently more environmentally friendly.

The authors have planned extensive research to conduct a comprehensive assessment of the suitability of molding sand technology with the new SH/PE lustrous carbon carrier for the foundry industry. They identified three primary research directions focused on (1) molding sand, (2) casting, and (3) ecology, which form the basis for the preparation of three parts of this publication.

This article evaluates the impact of the new SH/PE lustrous carbon precursor on the typical technological and mechanical properties of bentonite-bonded sands compared with additives already used in foundry practice (systems with hydrocarbon resins and carbon dust).

The second part of this study, covering the production of a trial casting (iron) and the subsequent evaluation of its surface quality, will be presented in the second part of this article. Additionally, the authors aim to address environmental protection and occupational safety in foundry operations. Therefore, the third part of this article will focus on a broader ecological assessment of the proposed solution.

## 2. Materials and Methods

### 2.1. Basic Materials

The mineral fraction in the form of sodium bentonite, commercially known as Specjal (S) (ZGM “Zębiec” S.A., Starachowice, Poland), was used during the research. The chemical composition and basic physicochemical properties of S bentonite are summarized in Table 1.

Table 2 lists the initial components used to prepare the green sand, which were subsequently sent for further testing.

### 2.2. Preparation of Molding Sand

The molding sand was prepared in a WADAP LM-1 circular mixer according to the recipe: 100 parts by weight of quartz sand, 6 parts by weight of binding material, and water added to obtain the required humidity. Detailed data on the proportions of the individual compositions are given in the summary table summarizing the composition of all the molding sands analyzed in this work (Table 3). The technological and mechanical properties of the molding sand were assessed depending on the water content; therefore, its share in the molding sand was not included in Table 3. The composition of the molding sand was determined based on the literature data [1,5,6].

First, the grain matrix (quartz sand) was mixed with bulk materials (bentonite, carbon carrier)—mixing time 1 min. Then, water was added and the whole was mixed for another 3 min. The resulting homogeneous molding sand in terms of moisture and composition throughout its entire volume was sieved through a 4 × 4 mm sieve. Due to the research on changes in the analyzed molding sand indices depending on their moisture (by adding water), each time the molding sand was returned to the mixer bowl, an appropriate portion of water was added and the mixing process was repeated for 3 min. The tests were carried out at an air humidity of about 29% and an ambient temperature of 23 to 26 °C.

Shungite was used as a new carbon additive in the field of foundry engineering, with its effectiveness and reduced environmental footprint confirmed in a patent application (invention project titled “Bentonite-bound molding sand with carbon additive”, Patent No: P.439688). Additionally, polyethylene (PE) was utilized as an additive, as it has a similar carbon content and is intended as a substitute for HCR resin. Four types of molding sands with carbon additives were prepared: the CD/HCR mixture was introduced into the first sand, the SH/HCR mixture into the second, the SH/PE mixture into the third, and the CD/PE mixture into the fourth sand. The amount of shungite was determined based on its carbon content compared with coal dust and HCR resin; thus, its mass proportion in the molding sands was proportionally higher than that of the other glossy carbon carriers. Table 3 summarizes the compositions of the prepared molding sands.

### 2.3. Preparation of Laboratory Samples

Standardized cylindrical shapes were made from the prepared foundry sand to determine their compressive strength (R_c_^w^) and tensile strength (R_m_^w^). Three cylindrical samples (⌀50 × 50 mm) were used for each specific property determination. Compaction was achieved by three blows of a LU-type laboratory rammer with a rammer weight of 6.66 kg dropped from a height of 50 mm.

### 2.4. Investigations of Green Sands

Mechanical tests of the molding sand were carried out using a universal device for determining mechanical properties, LRu-2e by Multi-serw Morek, in a wet sand. The determination of R_m_^w^ was performed by placing the sample vertically between the compression jaws, with a diameter corresponding to the diameter of the specimen’s base. An axial pressure was then applied to the sample until it fractured. The determination of R_c_^w^ was carried out by subjecting the sample to tensile force until it was destroyed. The measuring ranges were 0–22.4 N/cm^2^ for R_c_^w^ and 0–130 N/cm^2^ for R_m_^w^. The strength values were expressed in the SI unit of MPa. The tests were conducted according to the PN-83/H-11073/EN standard [37]. The measurement of tensile strength in the over-moistened zone (R_k_) was conducted on standard cylindrical samples. The sample, with a specified moisture content, was placed in the apparatus such that its front surface was positioned under a heating plate (heated to 320 °C). Once the sample was inserted into the guide, the plate automatically pressed down on it, providing heat. The heating time was experimentally determined to ensure that a saturated zone was created precisely at the plane of separation of the sample. After the heating period, the suction pump was automatically activated, causing the sample in the saturated zone to stretch until rupture occurred. The results of the measurement were recorded on a manometer calibrated in G/cm^2^.

The molding properties index (W_f_), determined according to the H.W. Dietert method, was assessed using a 200 g sample of molding sand that had been sieved through a 10 × 10 mm sieve. The molding sand was placed in a drum sieve inclined at an angle of 7° to the horizontal. The sieve had a diameter of 178 mm, featured holes sized at 2.4 mm, and was driven by an electric motor that rotated it at a speed of 0.95 rpm (equivalent to 57 rpm). The measurement lasted for 10 s, during which the molding sand that passes through the drum sieve was collected and weighed. The molding properties index (W_f_) was expressed as a percentage of the initial sample mass (200 g).

This determination was conducted for at least four different moisture levels, with three samples taken for each specified moisture level. The differences in results should not exceed 10% of the arithmetic mean value; otherwise, the entire measurement procedure must be repeated.

Permeability in a wet state state (P^w^) was determined using an electric device type LPiR1. The permeability values were expressed in the SI unit: m^2^/Pa·s. The determination was conducted for standard cylindrical samples according to the PN-80/H-11072 standard [38].

The compactibility (Z) was determined based on the measurement of the percentage reduction in the height of a column of the sand loosely poured into a metal tube under the influence of a constant pressing pressure.

The flowability (P_D_) of the molding sand was assessed according to the methods of H.W. Dietert and F. Valtier using a hand rammer equipped with a fixed sensor to measure the degree of deformation of standard cylindrical-shaped samples between the fourth and fifth blows of the standard hand rammer’s weight. It should be noted that none of the methods for testing the flowability of green sands have been standardized globally to date.

The numerical value of Dietert’s flowability, expressed in percentage (%), was determined using the following Formula (1) [1]:

P_D_ = 100 − 40*x* (1)

where *x*—the loss of height of the cylinder, mm.

The free flow (P_S_) method involves using a constant mass of foundry sand (150 g), irrespective of the moisture and binder content. The sample was sieved through a 4 × 4 mm sieve into a funnel-shaped container with an opening bottom. After sieving, the hook holding the bottom was released and the sand was allowed to fall by gravity from a height of 3 ft (0.914 m) onto a 6 × 6 mm sieve. The free flow (P_S_) is a measure of the amount of sand, expressed in grams, that passes through the sieve. This sand was weighed to the nearest 0.1 g. The more sand that passes through the sieve, the better the flowability.

The friability (S_LS_) tests were conducted using an LS apparatus (produced by Huta Stalowa Wola). The measurement consisted of determining the relative loss of mass of a cylindrical sample after rolling it over a pair of rollers with a diameter of 50 mm (at 750 rpm for 5 min) while being heated by an infrared lamp. The determination was carried out in accordance with the BN-77/4024-02 standard [39].

The apparent density ρ_0_, due to the pores between the grains, is always less than the density ρ and changes with the compaction of the molding sand. The determination of apparent density ρ_0_ was carried out by measuring the volume and mass of a cylindrical sample made from the tested molding or core sand. It is best to measure the height of the sample before removing it from the sleeve, using a caliper with a vernier scale to an accuracy of 0.1 mm. After removing the sample, its mass was weighed to an accuracy of 0.2 g, and then the apparent density ρ_0_ was calculated according to Formula (2) [1]:

(2) $$\rho_{0}=\frac{Q}{V_{c}};\;\mathrm{kg}/m^{3}\;(g/{\mathrm{cm}}^{3})$$

where Q—mass of the material (kg or g), and V_c_—volume of the material including pores (m^3^ or cm^3^).

## 3. Results

### Results of the Research and Their Discussion

**Figure 2:**
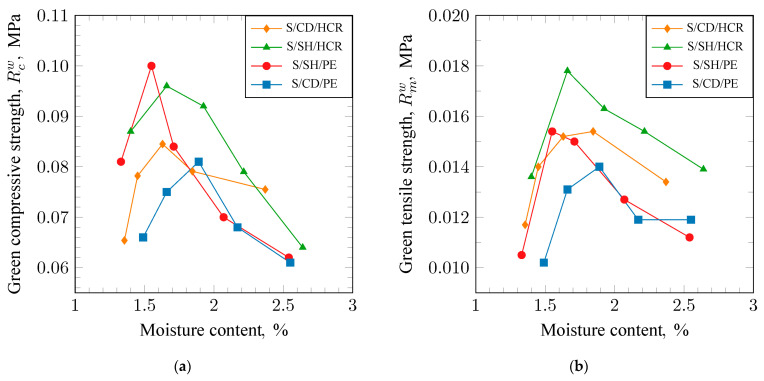
Green compressive (**a**) and green tensile strength (**b**) of molding sands bonded with sodium bentonite: S/CD/HCR, S/SH/HCR, S/SH/PE, and S/CD/PE.

The results of the strength measurements for the tested mixtures are presented in Figure 2a (R_c_^w^) and Figure 2b (R_m_^w^). The obtained trends of strength changes as a function of moisture content are similar. With increasing moisture, strength initially rises, and, after reaching a maximum, it decreases. For compressive strength R_c_^w^, the highest maximum value (0.10 MPa) was obtained for the S/SH/PE molding sand; this was slightly lower (0.096 MPa) for the S/SH/HCR molding sand, while the lowest value (0.08 MPa) was achieved for the S/CD/PE molding sand. The maxima of compressive strength for the individual mixtures were obtained at different moisture levels. The maximum was reached at the lowest moisture for the S/SH/PE molding sand, then at slightly higher moisture for the S/SH/HCR mixture, and at the highest moisture for the S/CD/PE molding sand. It can also be noted that the changes in strength for the S/SH/HCR molding sand show a gentle trend, which is advantageous because this mixture is not as sensitive to over-moistening as the others. A similar trend was observed in the case of tensile strength. However, in this case, a different arrangement for the maximum values can be seen. The highest value (0.018 MPa) of tensile strength was obtained for S/SH/HCR, a lower value (0.015 MPa) for S/SH/PE, and lowest (0.014 MPa) for S/CD/PE.

**Figure 3:**
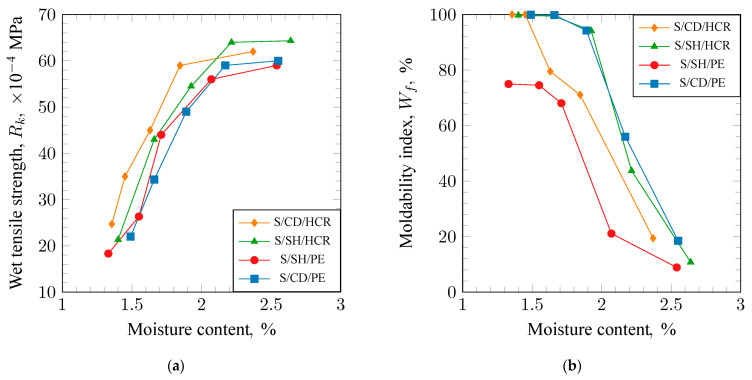
Welt tensile strength (**a**) and mobility index (**b**) of molding sands bonded with sodium bentonite: S/CD/HCR, S/SH/HCR, S/SH/PE, and S/CD/PE.

In the molds made from humid synthetic molding sands, a wet zone is created during the pouring of the casting alloy into the cavity of the mold, which can be the cause of surface defects in the casting. Therefore, these sands should exhibit the highest possible tensile strength in the wet zone, denoted as R_k_. The results of the measurements of this strength are presented in Figure 3a. An analysis of the obtained results allows us to conclude that the tested mixtures exhibited a similar trend in the changes of tensile strength R_k_ as a function of moisture content. The highest values across the entire examined range were obtained for the S/SH/HCR molding sand.

The determination of the molding property index W_f_ helps to establish the range of working moisture for the synthetic molding sand. This is the moisture level at which the mixture has optimal properties regarding its suitability for molding. It is assumed that the optimal working moisture occurs at a value of W_f_ = 75%. The results of the measurements of the molding property index for the examined compositions are presented in Figure 3b. An analysis of the obtained trends in W_f_ shows that the working moisture for the S/SH/PE molding sand is the lowest, at approximately 1.9%. In contrast, for the S/SH/HCR and S/CD/PE molding sands, this value is around 2.1%. When determining the range of working moisture, it is also necessary to consider the impact of moisture on the other properties of the mixture, particularly strength, permeability, and friability.

**Figure 4:**
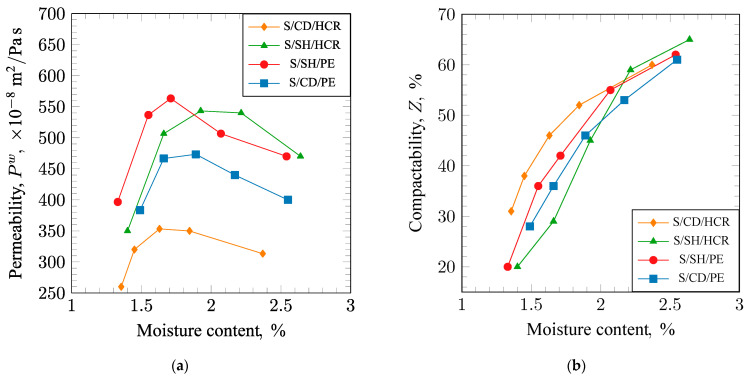
Permeability (**a**) and compactability (**b**) of molding sands bound with sodium bentonite: S/CD/HCR, S/SH/HCR, S/SH/PE, and S/CD/PE.

Figure 4 shows the results of determining the permeability (a) and compactability (b) of the analyzed green sands. The molding sand should exhibit good permeability to ensure the removal of gases from the mold cavity during the pouring of the liquid casting alloy. The results of the permeability measurements presented in Figure 3a indicate that each of the tested sands demonstrated very good permeability. As the moisture content of the sand increases, permeability rises, reaches a maximum, and then slowly decreases. Some differences can be observed in the trends of permeability changes with increasing moisture content for the individual sands. The highest permeability value (560 × 10^−8^ m^2^/Pa·s) was obtained for the S/SH/PE molding sand, while the lowest (470 × 10^−8^ m^2^/Pa·s) was obtained for the S/CD/PE molding sand. The maxima of permeability for the tested sands were achieved at different moisture levels, analogous to the minima of bulk density. The maximum for the S/SH/PE molding sand was reached at the lowest moisture content, at a slightly higher level for the S/CD/PE molding sand, and at the highest level for the S/SH/HCR molding sand.

The determination of compactability allows us to assess the suitability of the sand for molding and to determine whether the energy of the molding machine is being used optimally. The optimal value of this parameter should be around 40%. Analysis of the obtained results indicates that the trend of compactability changes with moisture content for the tested sand mixtures is very similar (Figure 4b). There is a shift between the curves towards higher moisture levels. Assuming the optimal compactability value is 40%, it can be observed that the S/SH/PE molding sand achieved this value at the lowest moisture content, the S/SH/HCR molding sand at the highest moisture content, and the S/CD/PE molding sand at an intermediate level. Such changes confirm the thesis that each sand requires a different moisture range to achieve optimal properties.

**Figure 5:**
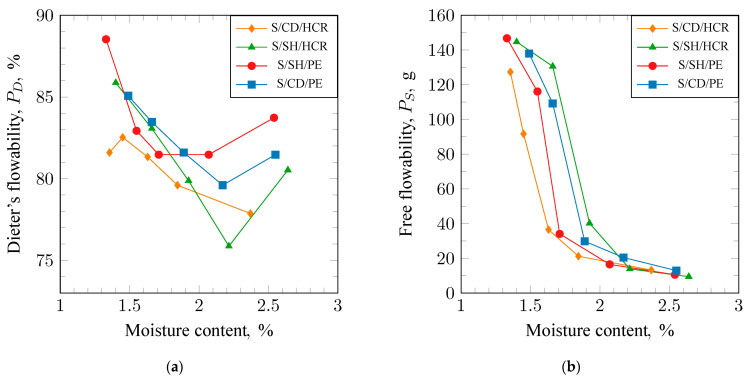
Dieter’s flowability (**a**) and free flowability (**b**) of molding sands bound with sodium bentonite: S/CD/HCR, S/SH/HCR, S/SH/PE, and S/CD/PE.

The flowability of the sand is the ability to achieve maximum and uniform compaction with minimal effort. This property determines the amount of work needed to obtain a well-compacted mold. Flowability was assessed using two methods. The results of the measurements are presented in Figure 5a (for Dietert flowability) and Figure 5b (for free flowability). Analysis of the results from the Dietert method indicated that the tested sand compositions exhibited similar trends in flowability as a function of moisture content. As moisture content increased, flowability decreased to a minimum and then increased again. The S/SH/PE molding sand achieved the highest value (81%) at the lowest moisture content. In contrast, the minima for the S/SH/HCR and S/CD/PE molding sands were obtained at higher, almost identical moisture levels, with values of 80% for S/CD/PE and 76% for S/SH/HCR, respectively.

Analysis of the results obtained using the free flowability method (Figure 5b) shows that the curves of flowability changes as a function of moisture content for the tested sands are also similar but shifted relative to each other. Such trends indicate that each sand has a different moisture range in which it achieves optimal properties.

**Figure 6:**
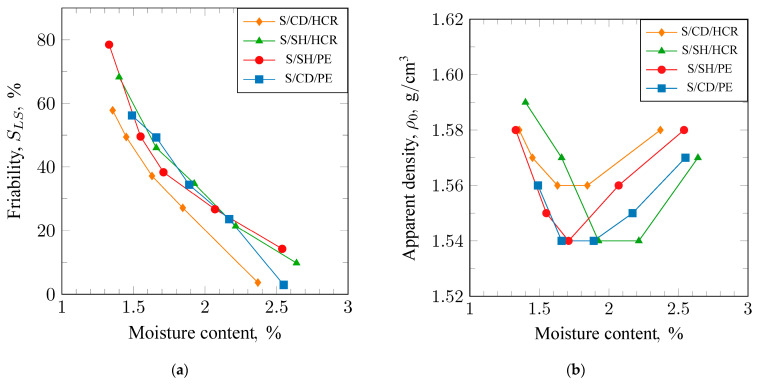
Friability (**a**) and apparent density (**b**) of molding sands bound with sodium bentonite: S/CD/HCR, S/SH/HCR, S/SH/PE, and S/CD/PE.

Flowability characterizes the ability of the molding sand to detach external grains from the surface of the mold cavity or core. The lower the flowability value, the less prone the material is to forming surface defects in castings associated with this, such as sand adhesion. The results of the flowability measurements conducted using the LS apparatus are presented in Figure 6a. It can be observed that the composition of the tested sands has minimal impact on flowability. The S/CD/PE molding sand performed the best in this regard, achieving a flowability value of approximately 3% at a moisture content of around 2.5%. The S/SH/HCR and S/SH/PE sands reached a flowability value of about 14% at this moisture level.

The results of the changes In apparent density with varying moisture content for sands of different compositions are shown in Figure 6b. It can be observed that an increase in moisture content initially leads to a decrease in apparent density, and, after surpassing the minimum, the density begins to increase. The trends obtained for the individual sands are similar, but there is a shift in the points of minimum density values. For the S/SH/PE molding sand, the minimum density was achieved at the lowest moisture content of approximately 1.7%. In the case of the S/CD/PE molding sand, a clear minimum density was not reached; however, the curve suggests that it will be lower than that of the S/SH/PE molding sand obtained at a slightly higher moisture content. The curve of apparent density changes for the S/SH/HCR molding sand is shifted towards higher moisture content compared with the trends for the S/SH/PE and S/CD/PE sands. In this case, a clear minimum density was also not achieved, but it is evident that its value will be the lowest and will occur at a moisture content above 2%. The samples for testing were prepared using a constant compaction effort, and the changes in apparent density indicate where the greatest resistance during compaction occurs.

## 4. Conclusions

The introduction of carbon precursors in the form of shungite and polyethylene (SH/PE) as substitutes for two popular carriers of graphite carbon, namely carbon dust and hydrocarbon resin, has resulted in synthetic molding sands with desired strength and technological properties. Both compressive strength and tensile strength meet the required values set for this group of sands across the entire range of tested moisture content. Additionally, all tested sands exhibited similar trends in the changes of tensile strength *R_k_*, Dietert flowability (P_D_), and free flowability (P_S_) as a function of moisture content. Notably, the sand containing SH/PE demonstrates the highest permeability and flowability. Furthermore, based on the analysis of the obtained results, it should be concluded that to achieve the desired level of the measured properties in the sand with the mixture of precursors SH/PE, the moisture content should be in the range of 1.5% to 1.7%.

The obtained results clearly indicate that the molding mixture incorporating the new SH/PE lustrous carbon precursor is suitable for use in foundry technology. Its properties are comparable to those of mixtures currently employed in the industry (comparison of the determined properties with the reference S/CD/HCR green sand). Thus, a scientific and practical foundation has been established to continue research on this system. For a comprehensive evaluation of the proposed solution, including its advantages and disadvantages, the research team proceeded to conduct a melting process and subsequent studies on the environmental impact and workplace safety associated with the technology.

The primary objective of introducing lustrous carbon carriers into bentonite molding mixtures is to prevent the burn-on defect of casting molds during pouring with molten iron. Due to the high dynamics of physicochemical processes occurring at the liquid metal–mold interface, the authors further focused on evaluating the surface quality of the castings, including surface roughness and dimensional accuracy. A description of the trial casting process (using iron) and a preliminary assessment of its surface quality will be presented in the second part of this article.

In the third part, an environmental assessment of the casting production process utilizing the technology developed by the research team will be conducted. The authors will present the results of gas emission studies from the mixture containing the new SH/PE lustrous carbon precursor (e.g., the kinetics of gas product release, changes in the volume of released gases, and BTEX emission levels). Finally, a method for recycling the spent molding mixture will be proposed. The ecological characterization will be discussed in comparison with data for industrially used lustrous carbon carriers (mixtures containing hydrocarbon resins and carbon dust).

## Figures and Tables

**Table 1:** Chemical composition and basic physicochemical parameters of sodium bentonite (S).

Chemical Composition		Montmorillonite (MMT) Content, %	Cation Exchange CapacityCEC, mmol/100 g	Swelling Index W_p_, cm^3^/2 g	Carbonate Content, %	Humidity, %	pH (10% r-r)
Oxide Composition	Content, %						
SiO_2_	79.6	79.6	75.1	35.4	5.0	9.3	10.1
Al_2_O_3_	18.50						
MgO	3.54						
CaO	3.06						
Fe_2_O_3_	5.22						
Na_2_O	3.37						
K_2_O	1.32						
Sum	98.90						

**Table 2:** List of molding sand ingredients.

Material	Role in the Molding Sand	Supplier	Technical Data/Properties
Silica sand	Sand grains (matrix)	Sibelco, Bukowno, Poland	Main fraction 0.16–0.32 mm
Hydrocarbon resin (HCR)	Carbon additive	ZGM Zębiec S.A, Starachowice, Poland	Carbon content (C): 98.5%; LC content *: min. 55%; Moisture content: max. 0.4%; Ash content: max. 0.5%; Softening temperature: 95–115 °C; volatile fraction content: 95.4%.
Coal dust (CD)	Carbon additive	ZGM Zębiec S.A, Starachowice, Poland	Carbon content (C): 97.5%; LC content *: min. 9%; Moisture content: max. 0.5%; Ash content: max. 5.6%; Volatile fraction content: max. 36.0%.
Shungite (SH)	Carbon additive	Wessper, Rzezawa, Poland	Carbon content (C) based on XRF: 77.1%; LC content *: 25%; Other elemental composition based on XRF: 11.7% O; 8.9% Si; 0.7% Al; 0.6% Fe; 0.4% K; 0.3% S; 0.1% Mg.
Polyethylene (PE)	Carbon additive	Hostalen, Bassel Orlen, Płock, Poland	Manufacturer’s proprietary data: High density; Density: 0.958 g/cm^3^; LC content *: 38%.

* Ability to create LC determined according to industry standards [36].

**Table 3:** Composition of green sand with bentonite and carbon additives (in part by weight; pbw).

Green Sand—Labelling at Work	Sand Grains (Matrix), pbw	Sodium Bentonite, pbw	HCR Resin, pbw	Coal Dust, pbw	Shungite, pbw	Polyolefin, pbw	Sum of Carbon Additives, pbw
S/CD/HCR *	100	4.5	0.56	2.79	-	-	3.35
S/SH/HCR	100	4.5	0.56	-	2.79	-	3.35
S/SH/PE	100	4.5	-	-	2.79	0.56	3.35
S/CD/PE	100	4.5	-	2.79	-	0.56	3.35

* Reference molding sand, used in the foundry industry.

## Data Availability

The data are contained within the article and/or are available on request from the corresponding author.
